# Discovery of a Novel Compound with Anti-Venezuelan Equine Encephalitis Virus Activity That Targets the Nonstructural Protein 2

**DOI:** 10.1371/journal.ppat.1004213

**Published:** 2014-06-26

**Authors:** Dong-Hoon Chung, Colleen B. Jonsson, Nichole A. Tower, Yong-Kyu Chu, Ergin Sahin, Jennifer E. Golden, James W. Noah, Chad E. Schroeder, Julie B. Sotsky, Melinda I. Sosa, Daniel E. Cramer, Sara N. McKellip, Lynn Rasmussen, E. Lucile White, Connie S. Schmaljohn, Justin G. Julander, Jeffrey M. Smith, Claire Marie Filone, John H. Connor, Yasuteru Sakurai, Robert A. Davey

**Affiliations:** 1 Departments of Microbiology and Immunology, University of Louisville, Louisville, Kentucky, United States of America; 2 Center for Predictive Medicine for Biodefense and Emerging Infectious Diseases, University of Louisville, Louisville, Kentucky, United States of America; 3 Department of Pharmacology and Toxicology, University of Louisville, Louisville, Kentucky, United States of America; 4 Drug Discovery Department, Southern Research Institute, Birmingham, Alabama, United States of America; 5 University of Kansas Specialized Chemistry Center, Lawrence, Kansas, United States of America; 6 The United States Army Medical Research Institute for Infectious Diseases, Ft. Detrick, Maryland, United States of America; 7 Institute for Antiviral Research, Utah State University, Logan, Utah, United States of America; 8 Boston University, Boston, Massachusetts, United States of America; 9 Texas Biomedical Research Institute, San Antonio, Texas, United States of America; The Scripps Research Institute, United States of America

## Abstract

Alphaviruses present serious health threats as emerging and re-emerging viruses. Venezuelan equine encephalitis virus (VEEV), a New World alphavirus, can cause encephalitis in humans and horses, but there are no therapeutics for treatment. To date, compounds reported as anti-VEEV or anti-alphavirus inhibitors have shown moderate activity. To discover new classes of anti-VEEV inhibitors with novel viral targets, we used a high-throughput screen based on the measurement of cell protection from live VEEV TC-83-induced cytopathic effect to screen a 340,000 compound library. Of those, we identified five novel anti-VEEV compounds and chose a quinazolinone compound, CID15997213 (IC_50_ = 0.84 µM), for further characterization. The antiviral effect of CID15997213 was alphavirus-specific, inhibiting VEEV and Western equine encephalitis virus, but not Eastern equine encephalitis virus. *In vitro* assays confirmed inhibition of viral RNA, protein, and progeny synthesis. No antiviral activity was detected against a select group of RNA viruses. We found mutations conferring the resistance to the compound in the N-terminal domain of nsP2 and confirmed the target residues using a reverse genetic approach. Time of addition studies showed that the compound inhibits the middle stage of replication when viral genome replication is most active. In mice, the compound showed complete protection from lethal VEEV disease at 50 mg/kg/day. Collectively, these results reveal a potent anti-VEEV compound that uniquely targets the viral nsP2 N-terminal domain. While the function of nsP2 has yet to be characterized, our studies suggest that the protein might play a critical role in viral replication, and further, may represent an innovative opportunity to develop therapeutic interventions for alphavirus infection.

## Author Summary

Alphaviruses occur worldwide, causing significant diseases such as encephalitis or arthritis in humans and animals. In addition, some alphaviruses, such as VEEV, pose a biothreat due to their high infectivity and lack of available treatments. To discover small molecule inhibitors with lead development potential, we used a cell-based assay to screen 348,140 compounds for inhibition of a VEEV-induced cytopathic effect. The screen revealed a scaffold with high inhibitory VEEV cellular potency and low cytotoxicity liability. While most previously reported anti-alphavirus compounds inhibit host proteins, evidence supported that this scaffold targeted the VEEV nsP2 protein, and that inhibition was associated with viral replication. Interestingly, compound resistance studies with VEEV mapped activity to the N-terminal domain of nsP2, to which no known function has been attributed. Ultimately, this discovery has delivered a small molecule-derived class of potent VEEV inhibitors whose activity is coupled to the nsP2 viral protein, a novel target with a previously unestablished biological role that is now implicated in viral replication.

## Introduction

Emergence and re-emergence of arboviruses such as alphaviruses continue to present serious health and economic threats [1], [2]. New World alphaviruses, family Togaviridae, including Venezuelan (VEEV), eastern (EEEV), and western (WEEV) equine encephalitis viruses, also represent significant biological defense threats, prompting these agents to be classified as Category B priority biodefense agents [3]. Most VEEV infections in humans are non-lethal, however, about 14% of the cases show acute disease symptoms affecting the central nervous system, resulting in fatalities in a small percentage of cases (<1%) [4]. Children are more susceptible to the neurological disease than adults. The lack of therapeutics for treatment, the possibility of accidental aerosol exposure of laboratory workers and its possible use as a bioterrorism agent highlight the importance of developing safe and effective anti-VEEV therapies.

Despite the urgent need, neither FDA-approved small molecule drugs or vaccines for VEEV are available. Two experimental VEEV vaccines, TC-83 and C84, have been developed for prophylaxis. The TC-83 vaccine is an attenuated virus derived from wild-type Trinidad donkey (TrD) strain (subtype IAB) by serial passage in tissue culture [5]. The TC83 vaccine has been provided as an investigational product to at-risk populations by the United States Army Special Immunizations program [6]. The C84 vaccine, which is inactivated VEEV TC-83, was developed to accommodate individuals who do not seroconvert after receiving the live TC-83 vaccine. Most individuals tolerate the C84 vaccine well, but the protection is short-lived and far less effective against aerosol challenge in hamster models than that of TC-83 [3]. Neither vaccine provides protection against an aerosol challenge in mice or nonhuman primates [7], [8]. Efforts to improve prophylactic vaccines for VEEV continue; however, a post-exposure therapeutic is a greatly needed alternative for sporadic outbreaks or from an intentional release.

Considerable research has been devoted to the discovery of new antivirals for VEEV infection. For many years, inosine-5′-monophosphate dehydrogenase inhibitors, such as ribavirin, VX-497 and mycophenolic acid, have been recognized to have antiviral activity in vitro [9]. Additionally, (-)-carbodine, a cytosine analogue, displayed anti-VEEV efficacy in vitro; however the in vivo efficacy was moderate [10]. Another reported VEEV inhibitor, a quinazolinone compound, has moderate activity against VEEV and Tacaribe virus (an Arenavirus), with an IC_50_ of 16.7 µM [11]. Most recently, the GSK-3β inhibitor, BIOder, was reported to decrease viral replication and pathogenesis from VEEV infection [12]. To our knowledge, none of these compounds have progressed past preclinical testing.

To find promising new leads for antiviral compounds for VEEV, we embarked on a high-throughput screening (HTS) campaign using the VEEV strain TC-83. Herein we present the discovery of a potent antiviral (CID15997213) showing promising antiviral activity *in vitro* and *in vivo* with low toxicity. Moreover, the CID15997213 targets the amino-terminal domain of the VEEV nonstructural protein 2 (nsP2), revealing a previously unrecognized biological function of this domain. This scaffold is a promising candidate for further optimization and preclinical testing for the development of anti-VEEV therapeutics.

## Results

### High-throughput screening and identification of hits

We screened a total of 348,140 compounds from the NIH Molecular Libraries Small Molecule Repository (MLSMR) library at a concentration of 20 µM with a Vero 76-based assay that measures cytopathic effect (CPE) from VEEV TC-83 infection. Prior to screening, the assay was standardized and validated for HTS. The average Z′ score during the screen was 0.84±0.04 (**Figure S1**). The ability of each compound to inhibit VEEV TC-83 CPE was measured three days post-infection. The cut-off for antiviral activity was 13.69% inhibition of CPE resulting in a 1.04% hit rate and 3,608 hits (**Figure S2**).

**Figure 1:**
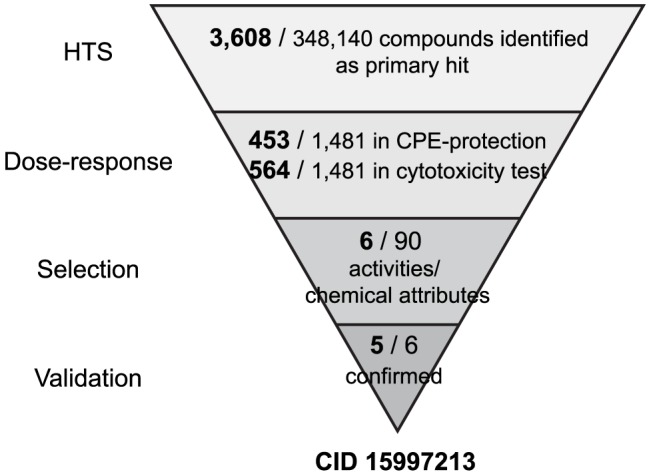
HTS of 348K compounds and identification of the hit compound. A flow diagram of various assays used in the screen. The number of hits remaining after each run is indicated in bold.

To down-select the hit compounds identified in the HTS, we used the CPE-based assay in a dose response format. Using a cheminformatic approach, we selected a total of 1,481 re-supplied compounds available from the MLSMR compound repository. The compounds were tested for potency in a ten-point dose-response format with concentrations that ranged from 0.5–25 µM. In parallel, we tested the compounds for cytotoxicity in the Vero 76. The dose-response experiment identified 453 compounds that showed >30% inhibition of VEEV TC-83 and acceptable dose-response activity profiles. The cytotoxicity test identified 564 compounds that showed >70% cell viability at all concentrations tested. Combined, the results highlighted 90 compounds that met our initial criteria: IC_50_ values <12.5 µM, CC_50_ values >25 µM and a Selectivity Index (SI) >2 (**Figure 1**).

**Figure 2:**
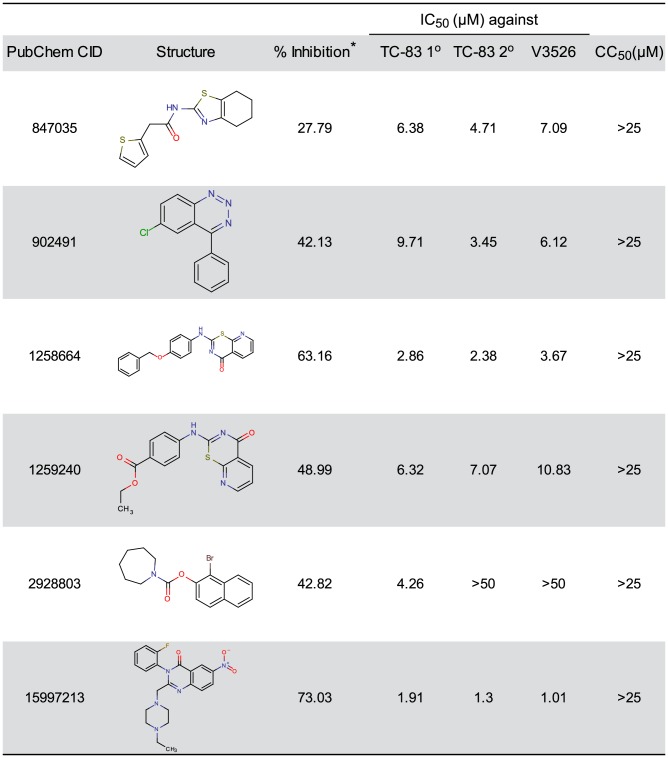
Selected small molecules with potent antiviral activity. * Percent inhibition at a concentration of 20 µM with the primary screening.

The 90 hit compounds identified by the dose-response assay were ranked on the basis of structure-activity-relationship (SAR) tractability -if SAR was present in the hit set-, lack of reactive or unstable functionality, synthetic feasibility, potency (IC_50_), and CC_50_. Additionally, a PubChem promiscuity analysis was assessed by the number of times the compound showed positive in distinct assays versus the number of times it was found to be active at concentrations less than 10 µM, and acceptable aqueous solubility in PBS for selected scaffolds. Based on these analyses, we selected six compounds (**Figure 2**), which were purified and analyzed for structural purity as solid powders. Purified compounds were then screened in secondary assays for confirmation as follows.

### Validation of antiviral activity of most promising compounds in secondary assays

Six of the most promising selected compounds (**Figure 2**) were tested in a 96-well format using the CPE-based assay with the VEEV TC-83 and VEEV V3526 to confirm their anti-VEEV activities. V3526 is a live-attenuated virus derived by site-directed mutagenesis from a virulent molecular clone of the TrD strain, V3000 [13], [14]. Five compounds showed promising anti-VEEV activity with IC_50_ values less than 6.5 µM and CC_50_>25 µM. The anti-V3526 activity of the compounds was comparable to that for TC-83 (**Figure 2**). CID15997213 was the most active compound with an IC_50_ of 1.3 and 1.01 µM for TC-83 and V3526, respectively; hence, we selected CID15997213 as the lead anti-VEEV inhibitory compound for further studies (**Figure 2**). This compound showed no cytotoxicity up to 50 µM (**Figure S3**). The average IC_50_ of CID15997213 from 17 independent tests for TC-83 was 0.84 µM with a standard deviation of 0.27 (**Table 1**).

**Table 1:** Spectrum of antiviral activity of CID15997213.

Viral family	Virus	Antiviral Activity (µM) *
*Togaviridae* New World alphavirus	VEEV TC-83†	0.84
*Togaviridae* New World alphavirus	VEEV V3526	0.67
*Togaviridae* New World alphavirus	VEEV TrD	0.38/0.48^§^
*Togaviridae* New World alphavirus	EEEV	>20
*Togaviridae* New World alphavirus	WEEV	10
*Togaviridae* Old World alphavirus	CHIKV	>50
*Filoviridae*	Ebola virus (Zaire)-GFP	>10
*Paramyxoviridae*	RSV	>50
*Poxviridae*	VACV-LREV	>20
*Rhabdoviridae*	VSV-EGFP	>20

IC_50_ measured in a cell-based CPE assay (µM) with triplicate data points for VEEV 3526, TrD, CHIKV and RSV. IC_50_ v*alue presented here for VEEV TC-83 is the mean from 17 independent experiments.

† Log difference in progeny virus titers between in the absence/presence of the compound at 5 µM was >6. 0.05 MOI of VEEV TC-83 was used for infection.

‡ IC_50_ measured in Neuro 2A cell line.

**Figure 3:**
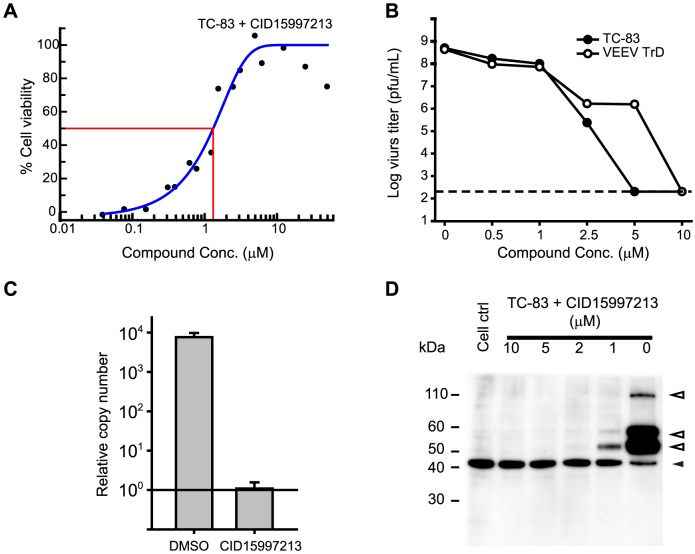
Anti-VEEV activity of CID15997213. (**A**) Dose response curve of CID15997213 in the CPE-based anti-VEEV TC-83 assay using Vero 76 cells from a representative experiment. (**B**) Titer reduction assay results for CID15997213. Vero 76 cells grown in 6-well plates were infected with 0.05 MOI of TC-83 or TrD and then incubated in the presence of CID15997213 at the denoted concentrations. Forty hours later the supernatant was harvested and the titer of the progeny virus was determined. Each point represents the mean from two independent experiments. The horizontal line in the figure indicates the detection limit of the assay. (**C**, **D**) Viral RNA and protein analysis. Vero 76 cells were infected with VEEV TC-83 at MOI of 5 and then incubated in the presence of DMSO or CID15997213 for 16 hours. In **C**, viral RNA was quantified using a quantitative real-time RT-PCR method with the total RNA from the cells. In **D**, proteins from VEEV TC83-infected cell extracts were analyzed by western blot assay. Closed triangles indicate bands corresponding to actin (internal control) and open arrows indicate bands that are specific to the viral proteins.

Additional confirmation of antiviral potency was measured for CID15997213 using plaque and titer reduction assays. Vero 76 cells were infected with 0.05 MOI of the TC-83 or TrD strain in the presence of the compound in the medium, and the titers of progeny viruses in the collected supernatant were measured in a microplaque assay. CID15997213 inhibited the replication of both viruses effectively in a dose-response manner (**Figure 3** ***B***). No progeny virus was detected at a concentration of 10 µM, indicating a complete inhibition of the replication of the viruses. In agreement, no visible plaques were developed when the TC-83 infected cells were overlaid with the agarose-overlay media containing CID15997213 at 5 µM (data not shown).

The final assays we used to confirm antiviral activity were real-time RT-PCR and western blot analyses of VEEV RNA (**Figure 3** ***C***) and proteins (**Figure 3** ***D***), respectively. Cells were infected at a MOI of 5 in the presence of CID15997213 and viral RNA and protein levels were measured 18 hours post-infection. The data showed that treatment with CID15997213 at a concentration of 5 µM inhibited viral replication resulting in a >8,000-fold reduction in viral RNA levels as compared to the control (**Figure 3** ***C***). In western blot analyses, treatment with the compound at 1 µM resulted in a dramatic decrease in viral protein level, and we were not able to detect viral proteins at compound concentrations higher than 2 µM.

### Spectrum of antiviral activity against wild-type VEEV, additional members of the *Togaviridae* and other virus families

To evaluate the spectrum of antiviral activity, we tested CID15997213 against representative viruses from additional members of the *Togaviridae* and other virus families in dose-response cell-based assays (**Table 1**). Antiviral activity of CID15997213 for VEEV TrD was equivalent to that of the TC-83 strain with IC_50_ values of 0.36 to 0.48 µM in Vero 76 or Neuro-2a cell lines. The SI for VEEV TrD in Vero 76 was 131. Two additional New World alphaviruses, Eastern equine encephalitis virus (FL91) and Western equine encephalitis virus (VR-70) showed no activity and a moderate activity (IC_50_ of 10 µM), respectively. Chikungunya virus, an Old World alphavirus, was not inhibited by CID15997213. No antiviral activity was observed against Ebola virus (Zaire), vesicular stomatitis virus, vaccinia virus (western reserve), or human respiratory syncytial virus (Long strain). There was no cytotoxicity associated with the compound in the cell lines at the concentrations tested. These results suggest that CID15997213 shows a selective and promising antiviral activity against VEEV and WEEV, albeit 10-fold lower, with minimal cytotoxicity.

### Mechanism of action studies

**Figure 4:**
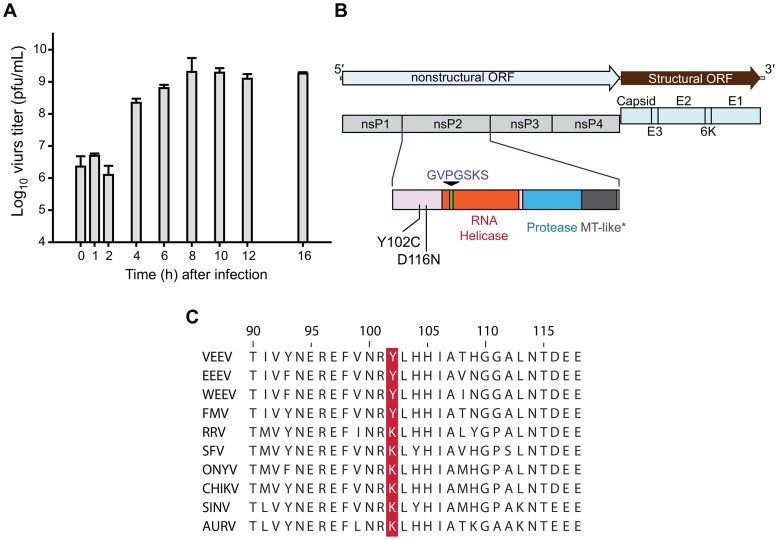
CID15997213 targets viral nsP2. (**A**) Time of addition study. Test compound, CID15997213, was added to the designated wells by replenishing the culture media with fresh culture media containing 5 µM of the compound at the time points denoted on the x axis. The graph denotes the virus titers at 16 hours post-infection from various time of addition points. Each data point is the mean from two independent replicates with duplication in titration. (**B**) Location of the mutations in the CID15997213 resistant viruses. The mutations mapped within the N-terminus of nsP2 protein (pink). There were no missense mutations in either nsP1, nsP3 or nsP4 genes. * Methyl-transferase like domain. (**C**) Sequence alignment of nsP2 alphaviruses. Amino acid sequences of nsP2 of following alphaviruses were aligned with Clustal W (www.clustal.org): VEEV (L01442.2, GenBank Access No. hereafter), EEEV (NC_003899), WEEV (NC_003908), Fort Morgan virus (FMV, NC_013528), Ross River virus (RRV, NC_001544), Semliki Forest virus (SFV, NC_003215), O'nyong-nyong virus (ONYV, NC_001512.1), CHIKV (L37661.3), Sindbis virus (SINV, NP_740671.1). Y102 position is highlighted in red.

To examine the point in the virus replication cycle at which the compound inhibits replication, we performed a time of addition experiment with CID15997213 (**Figure 4** ***A***) [15]. The addition of CID15997213 within 2 hours post-infection showed a similar level of activity as when added at time 0. However, the addition of the compound 4 hours post-infection lessens the antiviral activity and addition of the compound at 8 hours post-infection did not inhibit the replication of the virus at all. This suggests that CID15997213 targeted the virus during the middle stage of the virus's replication, rather than the early entry or later stages of assembly.

### Mapping of VEEV resistance mutations to CID15997213

To determine whether the CID15997213 inhibits VEEV directly, we asked whether resistance of the virus to the compound could arise. VEEV TC-83 was passaged with increasing concentrations of the compound (2.5 µM to 10 µM). At passage 4, virus began to emerge with resistance to the compound. Six plaques were purified from the eighth passage in the presence of CID15997213. The entire genome was amplified in 3 overlapping segments and sequenced (Table S2). As compared to the wild-type sequence, only two mutations were identified, Y102C and D116N, which are both located in nsP2 (**Figure 4B**). Five out of the six isolates carried Y102C mutation and one had D116N. VEEV TC-83 and V3526 harboring these mutations, Y102 and D116N, were completely resistant to CID15997213 with IC_50_ values greater than 25 µM in the CPE-based, dose-response assay (**Table 2**). The resistant viruses formed normal size plaques in the presence of the compounds at 5 µM (**Figure S4**); however, their peak virus titers were 10-fold lower than the parental virus strain (the median virus titers of 1.05×10^8^ pfu/mL vs. 3.9×10^9^ pfu/mL for wild type TC-83).

**Table 2:** Antiviral activity of CID15997213 with VEEV and VEEV mutants.

Virus	IC_50_ (µM)
TC-83	0.84
TC-83 Y102C	>25
TC-83 D116N	>25
V3526	0.32
V3526 Y102C	>25
V3526 D116N	>25

TC-83 Y102C and D116N were isolated by passaging of VEEV TC-83 in the presence of CID15997213. V3526 Y102C and V3526 D116N were generated by site-directed mutagenesis of pV3526 and virus was generated from synthetic RNA. The amino acid positions in the table refer to the position of the amino acid in the nsP2 protein.

To confirm the resistance of the mutant viruses, we used a reverse genetics approach. We introduced the Y102C and D116N mutations into the VEEV V3526 genome and tested the sensitivity of the rescued viruses, V3526 Y102C and V3526D116N, to CID15997213. Both strains showed complete resistance to CID15997213 (IC_50_>25 µM) while the parental strain, V3526 remained inhibited in the presence of compound (**Table 2**). This implies Y102 and D116N within the amino terminus of the nsP2 domain are targeted directly by CID15997213.

### *In vivo* efficacy of CID15997213 in a lethal VEEVTC83–mouse model

We first assessed the acute toxicity to define the maximum tolerated dose (MTD) of the CID15997213. The experimental design for the single dose range-finding study assessed four doses (1, 5, 50, 100 mg/kg) in one mouse per dose given by intraperitoneal administration (i.p.) at 0 hours. Mice were observed immediately after and for 24 hours for any adverse clinical signs. No mice showed any clinical signs suggesting no acute toxicity up to 100 mg/kg/day. In the multiple dose range-finding study of the CID15997213, three mice were used per dose (1, 5, 50, 100 mg/kg). In this study, each dose of CID15997213 was administered by i.p. twice daily on day (D) 0, 1, 2, and 3. Mice were examined twice daily for any adverse effects. No apparent toxicity of CID15997213 was shown in the mice at any of these concentrations as measured by body weight loss or any notable adverse effects as noted by potential for lethargy, hunched posture and ruffled fur. These studies suggest a MTD of CID15997213 in mice was ≥800 mg/kg.

Prior to testing for the efficacy of the compound in mice, we also conducted an *in vitro* absorption, distribution, metabolism, and excretion (ADME) study with CID15997213. CID15997213 showed 1) good blood-brain barrier (BBB) penetration potential, 2) low protein binding (32%) and 3) good microsomal and plasma stability (**Table S1**). These preliminary ADME studies suggested that the compound would have bioavailability.

**Figure 5:**
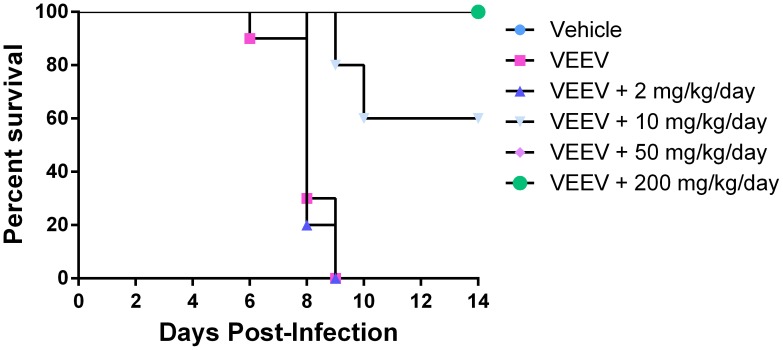
Effect of CID15997213 on survival of VEEV TC-83 infected mice. Six groups of ten C3H/HeN mice were used to assess antiviral activity *in vivo*; Group 1-Vehicle control; Group 2-VEEV only; Group 3- VEEV and 2 mg/kg/day CID15997213; Group 4- VEEV and 10 mg/kg/day CID15997213; Group 5- VEEV and 50 mg/kg/day CID15997213; Group 6- VEEV and 200 mg/kg/day CID15997213. Vehicle or CID15997213 were administered by i.p. four hours prior to mock or i.n. infection of VEEV TC-83. Treatments continued for from D0 though D5. Survival of mice in each group in plotted by time. Each group was compared to Group 3 for measurement of the p-value using the Mantel-Cox test. Analyses of each P value generated by the Mantel-Cox test were evaluated by comparison to a Bonferroni corrected threshold of 0.0125 (p = 0.05) to measure any potential significant differences between groups.

To screen for antiviral activity *in vivo*, we used the C3H/HeN mouse, which is susceptible to infection by the VEEV TC-83 strain and causes a lethal disease [16]. Based on the MTD and ADME data, we chose to evaluate for antiviral efficacy of CID15997213 at 2, 10, 50 or 200 mg/kg/day with 10 mice per group. One group received compound vehicle only (1% carboxymethylcellulose) and one group received virus only. Compound was administered two times per day by i.p. from D0 to D4. On D0, dosing started at 4 hours prior to virus challenge and 4 hours post-challenge. At 4 hours after the first administration of compound, mice were infected intranasally with 10 LD_50_ of TC-83. All animals were weighed and observed for clinical signs twice daily from the D0 through D14. The median time-to-death for the group challenged with VEEV was 8 days. The treatment of CID15997213 significantly increased survival of the groups treated with 50 or 200 mg/kg/day as compared to VEEV (**Figure 5**). There was no significant difference between VEEV group and 2 or 10 mg/kg/day

## Discussion

We report the discovery of a novel antiviral for VEEV with an excellent potency and safety profile from a large HTS. For HTS, we took advantage of the attenuated VEEV strain, TC-83 [5]. We developed a series of assays to screen and further evaluated the hits from a >340,000-compound library. We selected CID15997213, a quinazolinone compound as our top hit and confirmed its potency and lack of toxicity in several assays. Further, because VEEV is neurotropic, we assessed the antiviral activity of the compound in neuronal cells and found that the antiviral activity in the neuronal cells is almost identical to that observed in Vero 76 cell culture (**Table 1**). We also tested the CID15997213 compound for its spectrum of antiviral activity against several additional alphaviruses and viruses from other families. Neither EEEV nor CHIKV were inhibited in vitro by CID15997213. Broader spectrum screening also did not show any additional antiviral activity against Ebola virus, vesicular stomatitis virus, vaccinia virus, or human respiratory syncytial virus, suggesting selectivity for VEEV and WEEV.

Time of addition studies using VEEV TC83 in Vero 76 showed the compound had equivalent inhibitory activity if added as late as two hours post-infection. The effectiveness of the CID15997213 gradually decreased when added 4 hours post-infection and was not active at 8 hours post-infection. This suggests that the compound does not target entry or fusion, but a function required for replication of the virus. The replication cycle of alphaviruses progresses through three main stages: early, middle, and late, as defined by molecular synthesis. In the early stage of replication from entry until about 4 hours after, the nonstructural proteins (nsP123+nsP4) are translated from newly infecting genomic (+) RNA, and the (−) 42S RNA synthesis from the genomic (+) RNA takes place first, after which the (−) RNA are used as templates to make viral (+) RNA. The 42S (−) RNA synthesis shuts down approximately 4 hours after infection, correlating with the rapid cleavage of all nsP123 proteins into individual nsPs by protease activity in nsP2 [17]. During the middle stage of replication (4–8 hours post-infection), the viral replication complex synthesizes the 42S genomic (+) RNA and the 26S mRNA. Proteins are translated from the 26S mRNA and cleaved at this stage. In the final stage of replication, virions assemble at the plasma membrane and exit by budding. The maximum titer of virus in cell culture models is typically reached by16 hours [18], [19]. In summary, adding the drug at 4 hpi still allows 90% of the maximum virus production to occur suggesting the target of the compound is during minus strand synthesis.

A potential clue to the target during replication was provided by the compound resistance study, which identified two key resistance mutations in the N-terminal region of nsP2 (Y102C and D116N, **Table 2** **and** **Figure 4B**). The C-terminal regions comprising the helicase and the proteinase domains in nsP2 are well defined structurally or enzymatically [20]–[26]. The role of the N-terminus portion of nsP2, however, remains unclear. The nsP2 Y102 is conserved among VEEV, WEEV and EEEV, while the Old World alphaviruses have a K102 at this position (**Figure 4C**). This may explain the susceptibility difference between VEEV and CHIKV to the compound, but not among VEEV, WEEV and EEEV. Differences in the observed sensitivity of the VEEV, WEEV and EEEV may be due to differences in rates of viral replication. In addition to replication, nsP2 functions also include interacting with host functions in the nucleus and control of the host response and interferon production by the infected cells [27], [28]. This may also in turn effect viral replication. These aspects will be of interest for future studies of the mechanism of action of this drug.

We used the lethal VEEV TC-83-mouse model, which has been used widely for screening and efficacy studies of antivirals and vaccine candidates, to test the efficacy of CID15997213 [10], [16]. Forty percent of the challenged mice survived when treated with 10 mg/kg of CID15997213, and all mice survived when treated with 50 or 200 mg/kg/day. This efficacy is substantially better than other anti-VEEV compounds reported to date [10], [29], and hence shows promise for further development. For example, 100 mg/kg of (−)-carbodine was required to show 50% survival in the same model. Moreover, *in vitro* ADME studies suggest that CID15997213 was moderately effective at crossing the blood-brain barrier (BBB PAMPA, **Table S1**). Therefore, CID15997213 would be able to penetrate the blood brain barrier to fight infection.

In conclusion, we present the discovery of novel anti-VEEV compounds using a cell-based HTS of the MLSMR compounds library. This effort resulted in the identification of CID15997213, a potent hit compound, which is being optimized as a potential antiviral lead. In addition, the compound is being employed as a probe to study the role and the pharmacological relevance of intervening with the viral nsP2 domain. A comprehensive structure-activity study and mechanism of action study of the hit compound will be important to generate optimized lead compounds and develop a therapeutic candidate that can be used for the treatment of VEEV infection.

## Materials and Methods

### VEEV viruses and cells

VEEV TC-83 (lyophilized vaccine from USAMRIID) and V3526 were amplified in BHK-21 [30]. V3526 was rescued from the BHK cells transfected a full-length viral RNA derived from pV3526 plasmid as described elsewhere [31]. pV3526 was generated by removing the luciferase gene from pV3526-luc plasmid with QuickChange site-directed mutagenesis method (Stratagene). V3526 Y102C and V3526 D116N viruses were generated with same method from plasmids, pV3526 Y102C and pV3526 D116N, in which the corresponding mutation was introduced with QuickChange method. VEEV TrD (gift from Dr. R. Tesh, World Reference Center for Emerging Viruses and Arboviruses) and CHIKV (ATCC, VR-64) were grown in Vero 76 cells. (CRL-1587, ATCC) that were maintained in Dulbeccos-modified essential media (DMEM) with 10% FBS. BHK C-21 (CCL-10, ATCC). Neuro-2a (CCL-131, ATCC) were maintained in Minimum Essential Media with Earle's modification (MEM-E) with 10% FBS.

### VEEV HTS

348,140 compounds were plated in 384-well black wall plates containing 4,500 Vero 76 cells/well in single dose of 20 µM at a final concentration in Eagle's minimum essential medium with 5% heat inactivated FBS, 1% penicillin/streptomycin/L-glutamine, 1% Hepes and 0.2% DMSO. Twenty-five microliters of 175 TCID_50_ of VEEV TC-83 were added to each well using a Matrix WellMate. The plates were incubated for three days in an actively humidified incubator with 5.0% CO_2_ at 37°C and 95% humidity. The cell viability at the end of incubation period was measured as described elsewhere [32]. The Z factor values were calculated from 1 minus (3*standard deviation of cell control (σc) plus 3* standard deviation of the virus control (σv)/[mean cell control signal (μc) minus mean virus control signal (μv) [33].

### Antiviral dose response and cytotoxicity assays

1600 hits were selected for dose-response and cytotoxicity assays. Detailed procedures for these procedures are described in elsewhere (PubChem AID: 588727. http://pubchem.ncbi.nlm.nih.gov/assay/assay.cgi?aid=588727&loc=ea_ras).

A similar approach was used to measure the dose-response inhibition and cytotoxicity screens in a 96-well format against other viruses with a cell density of 12,000 cells per well in a volume of ninety microliters and 600 pfu of virus. Cell viability was measured with 90 µL per well of CellTiter-Glo reagent (Promega) after incubation for two days for VEEV or three days for CHIKV.

### VEEV microplaque, plaque and time of addition assays

To measure titer reduction using a microplaque assay, six well dishes with Vero 76 cells were infected with virus at an MOI of 0.05 in the presence or absence of media containing compounds. At 40 hours post-infection, the presence of PFU was measured as follows. Supernates from the 6 well plate from each treatment were diluted in DMEM supplemented with 5% FBS using a liquid handler, epMotion 5070 CB (Eppendorf Inc.). Vero 76 cells grown overnight in 96-well plates were infected with 25 µL of the serially diluted samples. The plates were incubated for 1 hour at 37°C, 5% CO_2_. Wells were rinsed with 100 µL of PBS and replenished with DMEM supplemented with 0.75% methylcellulose and 10% FBS and incubated at 37°C, 5% CO_2_ for three days. The microplaques were visualized by staining with 0.2% crystal violet in 4% paraformaldehyde and 20% ethanol.

For the plaque assays, six-well plates containing one-day-old Vero 76 cells were infected with 300 pfu of TC-83. After infection, an agarose overlay media (0.35% agarose, 1X MEM-E, 10% FBS) with or without CID15997213 (final concentration of 5 µM) was added. Plaques were visualized by 0.2% crystal violet in 4% paraformaldehyde.

For the time of addition assay, six-well plates containing Vero 76 cells were infected with 5 MOI of VEEV TC-83. At specific time before, during or after infection, 1.5 mL of cell culture media containing 10 µM of the compound was added. After 40 hours, supernate was harvested and PFU were measured.

### VEEV RNA quantitation

Total RNAs from infected cells were isolated with Trizol (Life Technologies) reagent as per the manufacturer's protocol and were dissolved in 50 µL of deionized water. Ten microliter of RNA samples were subjected to a cDNA synthesis with SuperScriptIII (Life Technologies) and random hexamers by following the manufacturer's protocol. To quantitate the relative viral RNA, we used a method of real-time PCR with 2(−Delta C(T)) method in conjunction with TaqMan chemistry. Sequences for the primers and probe are described in the **Table S2**. 18S rRNA was used for the endogenous control. The real-time PCR was done in a total of twenty microliters per well with 2 µL of 10-fold diluted cDNA mixture in a multiplex mode using ABI 9700HT genetic analyzer.

### Western blot

Western blot assay to detect viral protein was done with a standard protocol with anti-VEEV mouse monoclonal antibody 6 (DD-332, BEI Resources) and anti-actin rabbit polyclonal antibody (Sigma-Aldrich). The proteins were visualized with HRPO-conjugated anti-mouse IgG and HRPO-conjugated anti-rabbit IgG from goat in conjunction with an enhanced chemiluminescence substrate (GE Healthcare).

### VEEV resistance screening

CID15997213-resistant viruses were selected by passaging VEEV TC-83 in the presence of the compound. Drug concentration was increased by 2.5 µM every other passages starting at 2.5 µM (Passage 0) and ending at 10 µM (Passage 8). Resistant viruses were plaque-purified and amplified in the presence of the compound at 5 µM. Viral RNA was purified with MagMAX viral RNA isolation kit (Ambion) with 250 µL of virus culture supernatant. The nsP12, nsP34 and structural gene regions were amplified by RT-PCR with primers and PhusionTaq (NEB) (See the **Table S2**). The sequences were determined by standard automated Sanger sequencing and compared to the parental sequences (GenBank Accession No.: L01443.1) as reference.

### Broad spectrum CID15997213 screening

Compound CID15997213 was tested for antiviral activity using cell based assays for several viruses. For testing with EEEV strain FL91, Vero cells were infected for 1 hour at MOI = 0.05. Virus was then removed by washing and media was added with a 2-fold dilution of compound from 20 to 2.5 µM. Titers were assayed from supernatant collected at 40 hours post-infection by neutral red plaque assay. Compound was tested for activity against WEEV (VR-70, ATCC) in Vero cells (CCL-81, ATCC). Briefly, serial two-fold dilutions of compound from 25 to 0.2 µM were added to cells in a 96-well microplate followed by addition of virus. Cytopathic effect was evaluated by neutral red assay and visual observation on day 3 to determine the IC_50_. For measurement of potential inhibition of Ebolavirus infection of HeLa cells, a recombinant Ebolavirus with a green fluorescent protein (GFP) gene inserted into the genome was used. Cells were pretreated for 1 hour with two fold-dilutions of 10 to 0.005 µM of compound and incubated with virus for 24 hours in the presence of the compound. Fixed cells were imaged by microscope. Total and infected cells were counted by Cell Profiler image analysis software (Broad Institute, MIT, Boston, MA), detecting nuclei stained with DAPI and virus encoded GFP expression. This work was performed in a biosafety level 4 (BSL4) laboratory at Texas Biomedical Research Institute. Antiviral activity was tested against VACV (VACV-LREV) in A549 cells (CCl-85, ATCC). Briefly, serial dilutions of compound from 20 to 0.1 µM were added to cell in a 96-well microplate followed by addition of virus. Viral infection was assayed by the measurement of fluorescent reporter proteins from early (venus) or late (mCherry) VACV promoters 18 hours post infection to determine the 50% effective concentration (EC50) [34]. Compound was tested for activity against VSV-EGFP in A549 cells (CCL-85, ATCC). Briefly, serial dilutions of compound from 20 to 0.1 µM were added to cell in a 96-well microplate followed by addition of virus. Viral infection was assayed by the measurement of the fluorescent reporter protein eGFP at 18 hours post infection to determine the IC_50_ [35]. Antiviral activity against RSV was tested as described elsewhere [36]. Briefly, RSV (strain Long, ATCC VR-26) was amplified in HEp-2 cells grown in MEM-E and the RSV stock viruses were supplemented with 10% trehalose and then stored in vapor phase of liquid nitrogen [37]. Serially diluted compound from 25 to 0.2 µM were added to HEp-2 cells in a 96-well microplate followed by addition of virus (MOI = 0.05). CPE was evaluated by CellTiter-Glo reagent (Promega) after incubation for five days.

### Synthesis of quinazolinone CID15997213

CID15997213 is commercially available from ChemDiv, Inc. (CAS# 900134-28-3); however, it was synthesized at the University of Kansas for the purpose of scale, purification, structural analysis and purity confirmation prior to assessment in assays. CID15997213 was prepared by the route depicted in **Text S1**.

### *In vivo* dose-range finding and efficacy studies

This study was carried out in strict accordance with the recommendations in the Guide for the Care and Use of Laboratory Animals of the National Institutes of Health. The protocol was approved by the Institutional Animal Care and Use Committee of the University of Louisville (Protocol Number: 12011). All efforts were made to minimize pain and suffering. For single-dose and multiple dose range-finding studies, we assessed potential toxicity at four doses (1, 5, 50, 100 mg/kg) in mice. In single-dose, mice were examined at 0 and 24 hours after intraperitoneal administration in one mouse per dose. In the multiple dose range-finding study, three mice were used per dose (1, 5, 50, 100 mg/kg). CID15997213 was administered by i.p. twice daily for 4 days on day 0, 1, 2, and 3. Mice were weighed and examined twice daily for any adverse effects.

For antiviral screening for efficacy, ten five to six week old C3H/HeN mice obtained from Charles River Laboratories (Wilmington, MA) were randomly assigned to one of 6 treatment groups: Group 1-Vehicle control; Group 2-VEEV only; Group 3- VEEV and 2 mg/kg/day CID15997213; Group 4- VEEV and 10 mg/kg/day CID15997213; Group 5- VEEV and 50 mg/kg/day CID15997213; Group 6- VEEV and 200 mg/kg/day CID15997213. Mice were dosed twice per day with 200 µL volume comprised of vehicle only (1% carboxymethyl cellulose) or compound formulated in vehicle by i.p. Treatments were conducted for five days, beginning 4 hours prior to virus challenge. Mice were infected i.n. with 10 LD_50_ of TC-83 (Day-0) diluted in 50 µL of PBS. For the Vehicle control, PBS was used in place of virus. Mice were weighed from D0–D14 and checked twice a day for mortality and morbidity. P values were generated from comparisons of survival data using the Log-Rank (Mantel-Cox) test using Prism 6 (Graph Pad Software, Inc) and compared using the Bonferroni method. P values were calculated for each group (K = 4). Analyses of each P value generated by the Mantel-Cox test were evaluated by comparison to a Bonferroni corrected threshold of 0.0125 (p = 0.05) to determine significance.

## Supporting Information

Figure S1 **The primary HTS assay performance.** Z′ analysis (the average Z′ = 0.84) showed that the HTS was robust. Each data point represents the Z′ (see below) of each 384-well assay plate in the HTS.
(DOCX)

Figure S2 **Hit compounds selection from the HTS.** A total of 348,140 compounds were screened in the CPE based HTS at 20 µM. The average inhibition was 2.18%. The 3,608 compounds that showed an inhibition efficacy higher than the cut-off, 13.69% (mean +3 times of standard deviation of all compounds tested; shown by black horizontal line) were selected as hit compounds.
(DOCX)

Figure S3 **Cytotoxicity assay of CID 15997213.** CID 15997213 didn't show cytotoxicity in Vero 76 cells. Each data point represents the mean of percent cell viability from triplicates. Dose-Response curve and IC_50_ were generated using the Four Parameter Logistic Model or Sigmoidal Dose-Response model.
(DOCX)

Figure S4 **Plaques from resistant mutant viruses.** Viral plaques of TC-83 P8 which was selected by CID 15997213 treatment for 8 passages were developed in the presence of 5 µM of CID 15997213 (bottom). Even with the treatment of the compound, the size of plaques of the mutants (bottom) was nearly the same as that of wild type TC-83 produced in the absence of the compound (top).
(DOCX)

Table S1 ***In vitro*** **ADME profile of CID 15997213.**
(DOCX)

Table S2 **Sequences of primers and probes used for the experiments.**
(DOCX)

Text S1 **Synthetic route of CID 15997213.**
(DOCX)
