# Supplementary figures and images for: Discovery of a Novel Compound with Anti-Venezuelan Equine Encephalitis Virus Activity That Targets the Nonstructural Protein 2
Source: PLoS Pathog. 2014 Jun 26;10(6):e1004213. doi: 10.1371/journal.ppat.1004213 (PMC4072787; doi:10.1371/journal.ppat.1004213)

**Figure S1. The primary HTS assay performance.**

**
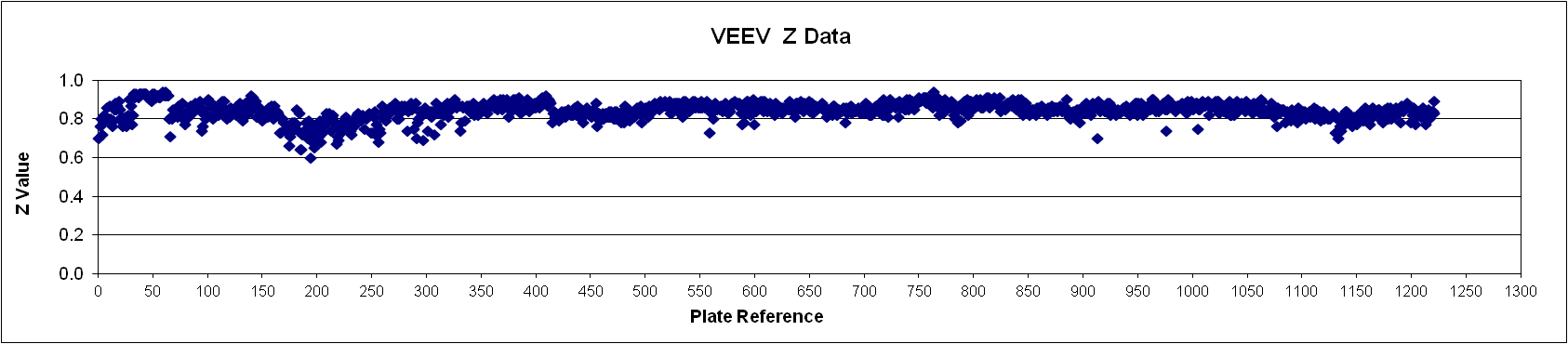
**

Supplement: Figure S1 — The primary HTS assay performance. Z′ analysis (the average Z′ = 0.84) showed that the HTS was robust. Each data point represents the Z′ (see below) of each 384-well assay plate in the HTS. (DOCX) [file ppat.1004213.s001.docx]

**Figure S2. Hit compounds selection from the HTS.**


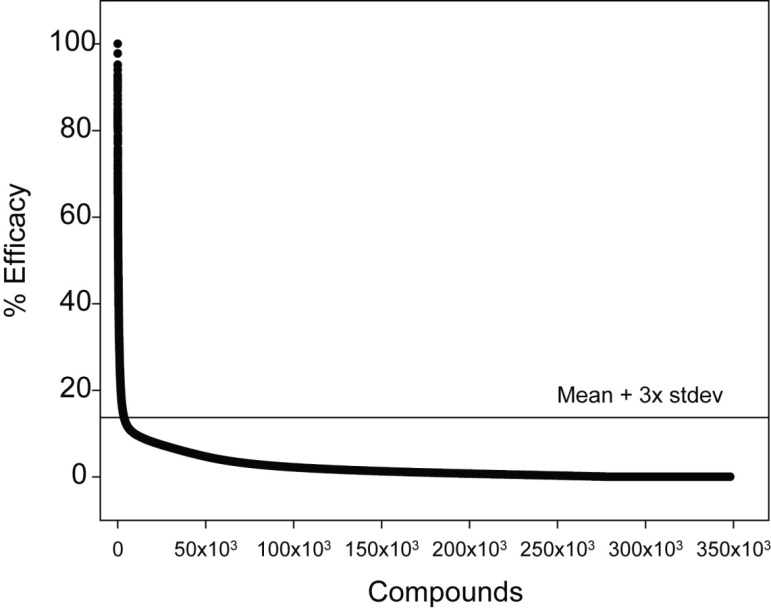

Supplement: Figure S2 — Hit compounds selection from the HTS. A total of 348,140 compounds were screened in the CPE based HTS at 20 µM. The average inhibition was 2.18%. The 3,608 compounds that showed an inhibition efficacy higher than the cut-off, 13.69% (mean +3 times of standard deviation of all compounds tested; shown by black horizontal line) were selected as hit compounds. (DOCX) [file ppat.1004213.s002.docx]

**Figure S3.** **Cytotoxicity assay of CID15997213.**


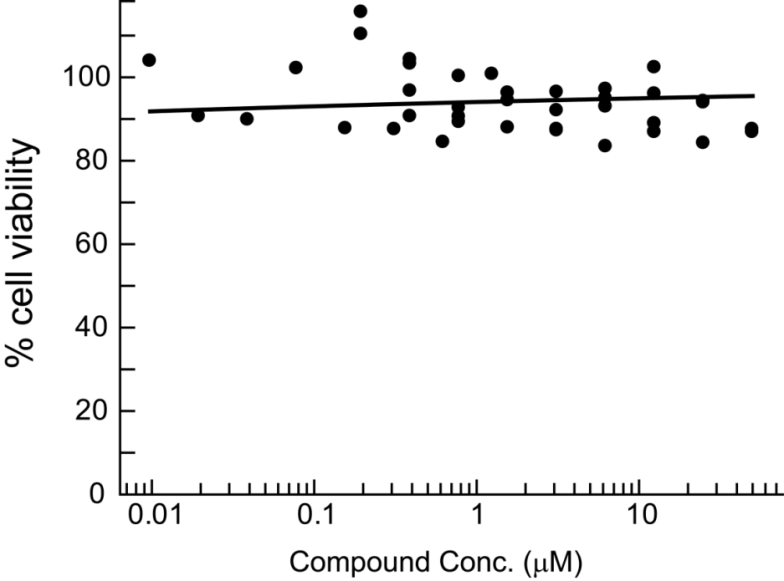

Supplement: Figure S3 — Cytotoxicity assay of CID 15997213. CID 15997213 didn't show cytotoxicity in Vero 76 cells. Each data point represents the mean of percent cell viability from triplicates. Dose-Response curve and IC50 were generated using the Four Parameter Logistic Model or Sigmoidal Dose-Response model. (DOCX) [file ppat.1004213.s003.docx]

**Figure S4. Plaques from resistant mutant viruses.**


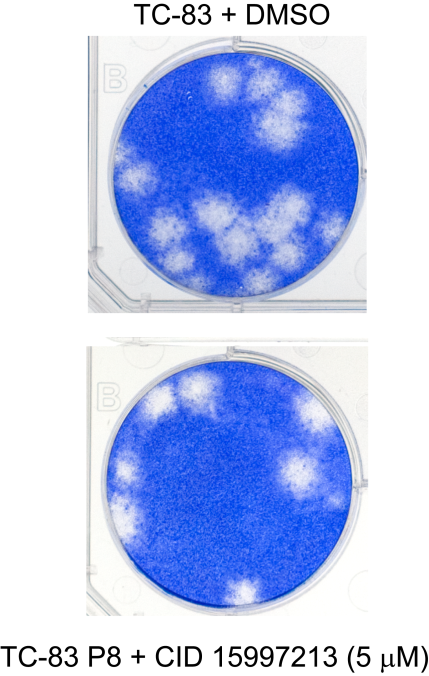

Supplement: Figure S4 — Plaques from resistant mutant viruses. Viral plaques of TC-83 P8 which was selected by CID 15997213 treatment for 8 passages were developed in the presence of 5 µM of CID 15997213 (bottom). Even with the treatment of the compound, the size of plaques of the mutants (bottom) was nearly the same as that of wild type TC-83 produced in the absence of the compound (top). (DOCX) [file ppat.1004213.s004.docx]
