# Supplementary material for: Discovery of a Novel Compound with Anti-Venezuelan Equine Encephalitis Virus Activity That Targets the Nonstructural Protein 2
Source: PLoS Pathog. 2014 Jun 26;10(6):e1004213. doi: 10.1371/journal.ppat.1004213 (PMC4072787; doi:10.1371/journal.ppat.1004213)
Supplement: Table S1 — In vitro ADME profile of CID 15997213. (DOCX) [file ppat.1004213.s005.docx]

Table S1. *In vitro* ADME profile of CID 15997213

| Aqueous solubility (µM) | | Mouse plasma half-life  T_1/2_^a^ | Microsomal intrinsic clearance  CL_int_^b^, T_1/2_^a^ | | Mouse plasma protein binding | | MDCK-MDR1 permeability ^c^ | | | BBB PAMPA  permeability  (10^-6^ cm/s) |
| --- | --- | --- | --- | --- | --- | --- | --- | --- | --- | --- |
| 45 min. | 16 hr. |  | NADPH- dependent | NADPH-free | Mean plasma fraction unbound/bound | Recovery (%) | mean A->B | mean B->A | Efflux ratio^d^ |  |
| >100 | >100 | >240 | 31.4,  73.5 min. | 0.0,  >240 min. | 32.4%/67.6% | 89.0% | 7.2 | 38.5 | 5.3 | 42 |
