# Supplementary material for: Discovery of a Novel Compound with Anti-Venezuelan Equine Encephalitis Virus Activity That Targets the Nonstructural Protein 2
Source: PLoS Pathog. 2014 Jun 26;10(6):e1004213. doi: 10.1371/journal.ppat.1004213 (PMC4072787; doi:10.1371/journal.ppat.1004213)
Supplement: Table S2 — Sequences of primers and probes used for the experiments. (DOCX) [file ppat.1004213.s006.docx]

**Table S2.** **Sequences of primers and probes used for the experiments**

| **Name** | **Sequences** | **Note** |
| --- | --- | --- |
| VEEV QRT probe | /56-FAM/TACGACATC/ZEN/GACAGGAAACAGTGCGT/3IABkFQ/ | Realtime PCR probe |
| VEEV QRT primer_fwd | AGGAGCGCTGAACACTGATGAAGA | Realtime PCR forward primer |
| VEEV QRT primer_rev | AGGCGAATTCATGGAAGGGAGGAT | Realtime PCR reverse primer |
| TC-83 1_fwd | ATGGGCGGCGCATGAGAGAAG | ns12 region PCR amplification |
| TC-83 4060_rev | TCCCCTCGCACCACATGATATGAGG | ns12 region PCR amplification |
| TC-83 4002_fwd | ACAGGTTCCAGACTCCACGAAGC | ns34 region PCR amplification |
| TC-83 7550_rev | AGACTATGTCGTAGTCCATTCAGG | ns34 region PCR amplification |
| TC-83_7532_fwd | ATGGACTACGACATAGTCTAGTCCG | Structural genes PCR amplification |
| TC-84_11421_rev | GAAATATTAAAAACAAAATCCGATTCG | Structural genes PCR amplification |
| VEEV_seq_309 | ACTTATACAATCTGTCCGGATC | sequencing primer (reverse) |
| VEEV_seq_603 | AACTTGGCTGGAGCATATCC | sequencing primer |
| VEEV_seq_1208 | CCAGGCATTTGCTAGGTGGG | sequencing primer |
| VEEV_seq_1826 | TTATGCCGTGGAACCATACC | sequencing primer |
| VEEV_seq_2423 | TGCAGGTACTCTCAGAGCGC | sequencing primer |
| VEEV_seq_3030 | TTGGAGAGACCGGACCCTAC | sequencing primer |
| VEEV_seq_3628 | GAGCTCGGCTGGATTTAGGC | sequencing primer |
| VEEV_seq_4209 | GAATGATATGTTTAGCTGCACC | sequencing primer (reverse) |
| VEEV_seq_4229 | TCATGCCGTAGGACCAAACT | sequencing primer |
| VEEV_seq_4832 | CTTGTGCATCCATGCCATGA | sequencing primer |
| VEEV_seq_5546 | AGAGGAGCTCGAGGCGCTTA | sequencing primer |
| VEEV_seq_6158 | CGGAGCTTCATGCTGCTTAG | sequencing primer |
| VEEV_seq_6759 | GACGCTATTATAGCCGAGCA | sequencing primer |
| VEEV_seq_7366 | GCTGGAACCGAGTGGGTATT | sequencing primer |
| VEEV_seq_7957 | GGAAGGGAAGATAAACGGCT | sequencing primer |
| VEEV_seq_8568 | CCGAGGAGCTGTTTAAGGAGTA | sequencing primer |
| VEEV_seq_9174 | GCACAAAGATCTCCGAGACC | sequencing primer |
| VEEV_seq_9765 | GGCTAACACCTAACGCTAGGATAC | sequencing primer |
| VEEV_seq_10367 | AAAGCGCACACAGCCTCAGT | sequencing primer |
| VEEV_seq_10984 | CGCAGTCCATGTGCCATCAG | sequencing primer |
| 1954_Y2C_rev | CACCTGTTTACGAACTCACGTTCGTT | In vitro mutagenesis (Y102C) |
| 1955_muta_fwd | CCTGCACCATATTGCCACACATGG | In vitro mutagenesis (Y102C) |
| 1995_D2N_fwd | AATGAAGAATATTACAAAACTGTCAAGCCC | In vitro mutagenesis (D116N) |
| 1994_muta_rev | AGTGTTCAGCGCTCCTCCATGTGTGGCAA | In vitro mutagenesis (D116N) |
