# Supplementary material for: Discovery of a Novel Compound with Anti-Venezuelan Equine Encephalitis Virus Activity That Targets the Nonstructural Protein 2
Source: PLoS Pathog. 2014 Jun 26;10(6):e1004213. doi: 10.1371/journal.ppat.1004213 (PMC4072787; doi:10.1371/journal.ppat.1004213)
Supplement: Text S1 — Synthetic route of CID 15997213. (DOCX) [file ppat.1004213.s007.docx]

**Synthetic route of CID15997213.**

Anthranilic acid **1** was treated with 2-chloroacetyl chloride in the presence of triethylamine to afford 2-(chloromethyl)benzoxazinone **2**. Dehydrative amidation was carried out by treating **2** with 2-fluoroaniline and POCl_3_[1]. The resulting 2-(chloromethyl)phenylquinazolinone **3** was aminated with N-ethylpiperazine to provide CID15997213 **4** in 31% overall yield over three steps.





**References**

1. STOCKWELL B (2009) COMPOUNDS AND COMPOSITIONS THAT CAUSE NON-APOPTOTIC CELL DEATH AND USES THEREOF. WO Patent 2,009,108,384.
